# Our First Number

**Published:** 1871-01

**Authors:** 


### OUR FIRST NUMBER.

A few hundred copies of this number will be sent to friends and others who have not yet become subscribers, but who, we hope, will soon—very soon.

We have already received many expressions of good will and words of cheer—many flattering compliments and encouraging promises. Nothing more certainly incites one to nobler and higher aspirations than a knowledge that his previous acts and efforts have been appreciated.

We intend to continue to advocate the truth, and sustain the law, and so far as we are capable, to make our Journal all that is expected of it by the profession. We offer no apology in occupying so much of this number in editorial matter. We could not say less and do justice to our opinions, our cause, and to those for whose interest it will be our pleasure to labor.

The Editors are alone responsible for the medical, biographical and hygienic department of the "Companion." The Publisher, Mr. J. J. Toon, is responsible only for the business department, embracing remittances, advertisements, and the mechanical execution of the Journal.

